# *Bartonella* spp. and Typhus Group Rickettsiae among Persons Experiencing Homelessness, São Paulo, Brazil

**DOI:** 10.3201/eid2902.221050

**Published:** 2023-02

**Authors:** Álvaro A. Faccini-Martínez, Louise Bach Kmetiuk, Lucas S. Blanton, Laís Giuliane Felipetto, Mara Lúcia Gravinatti, Jorge Timenetsky, Luiz Ricardo Gonçalves, Rosangela Zacarias Machado, Marcos Rogério André, Fabiano Borges Figueiredo, Andrea Pires dos Santos, Marcelo B. Labruna, Gustavo Monti, Alexander Welker Biondo, David H. Walker

**Affiliations:** Fundación Universitaria de Ciencias de la Salud, Servicios y Asesorías en Infectología, Hospital Militar Central, Bogotá, Colombia (Á.A. Faccini-Martínez);; University of Texas Medical Branch, Galveston, Texas, USA (Á.A. Faccini-Martínez, L.S. Blanton, D.H. Walker);; Oswaldo Cruz Foundation, Curitiba, Brazil (L.B. Kmetiuk, F.B. Figueiredo);; Federal University of Paraná, Curitiba (L.G. Felipetto, A.W. Biondo);; Central Paulista University Center, São Carlos, Brazil (M.L. Gravinatti);; University of São Paulo, São Paulo, Brazil (M.L. Gravinatti, J. Timenetsky, M.B. Labruna);; São Paulo State University, Jaboticabal, Brazil (L.R. Gonçalves, R Z. Machado, M.R. André);; Purdue University, West Lafayette, Indiana, USA (A.P. dos Santos);; Wageningen University and Research, Wageningen, the Netherlands (G. Monti)

**Keywords:** *Bartonella*, *Rickettsia*, bacteria, parasites, vector-borne infections, zoonoses, homelessness, São Paulo, Brazil

## Abstract

Persons experiencing homelessness in São Paulo, Brazil, were seropositive for *Bartonella* spp. (79/109, 72.5%) and typhus group rickettsiae (40/109, 36.7%). *Bartonella quintana* DNA was detected in 17.1% (14/82) body louse pools and 0.9% (1/114) blood samples. Clinicians should consider vectorborne agents as potential causes of febrile syndromes in this population.

Persons experiencing homelessness might be predisposed to vectorborne infections because of increased exposure to ectoparasites (*1*). Members of the genera *Bartonella* and *Rickettsia*, particularly the louseborne pathogens *B*. *quintana* and *R*. *prowazekii*, are agents of emerging illnesses among persons who are marginalized or experiencing homelessness (*1*). Studies on *Bartonella* and *Rickettsia* spp. infections in homeless populations within Latin America are scarce (*2*,*3*). Infestations with *Pediculus humanus humanus* body lice were reported in persons experiencing homelessness in Curitiba and São Paulo, 2 major cities in Brazil (*4*). We report results of molecular testing of lice and blood from persons experiencing homelessness in the city of São Paulo in southeastern Brazil. We evaluated their possible exposure to *Bartonella* spp. and typhus group rickettsiae (TGR) by using indirect immunofluorescence assays (IFAs). In addition, we assessed risk factors related to serologic status.

## The Study

During June–August 2018, a total of 114 persons experiencing homelessness (101 men, 13 women; average age 42.5 ±13.4 years) from a day-shelter in the city of São Paulo signed written informed consent forms and participated in this study, which was approved by the National Ethics Committee in Human Research (protocol no. 80099017.3.0000.0102). Persons responded to a questionnaire that, combined with medical and demographic records (Appendix), we used to assess risk factors. We carefully examined personal clothing and found lice in 14.9% (17/114, 95% CI 6.9%–19.7%) of persons; the lice were taxonomically identified as *P. humanus humanus* (*5*).

We analyzed 109 serum samples from study participants by using IFA to detect IgG against *Bartonella* spp. and TGR. We used commercial slides for *B. quintana* (12-well IFA Substrate Slides; Fuller Laboratories, http://www.fullerlaboratories.com) and in-house slides for *B*. *henselae* sequence type 9, *B*. *machadoae* 56A, *R*. *typhi* Galveston, and *R*. *prowazekii* Breinl strains. We found 79/109 (72.5%, 95% CI 63.1%–80.1%) persons were seropositive for *Bartonella* spp. and 40/109 (36.7%, 95% CI 27.7%–46.5%) were seropositive for TGR (titers >64). All antibody titers were >128 (Appendix Table 1), except for 2 *B. quintana–*positive and 8 TGR-positive samples. An endpoint titer >4-fold higher for a particular *Bartonella*/*Rickettsia* spp. antigen than that observed for other *Bartonella*/*Rickettsia* spp. antigens was considered the possible antigen involved in a homologous reaction (PAIHR) (*6*). Thus, *B. quintana* was the PAIHR in 75/79 (95.0%, 95% CI 87.5%–98.6%) persons, *R. typhi* was the PAIHR in 13/40 (32.5%, 95% CI 18.6%–49.1%) persons, and *R. prowazekii* was the PAIHR in 3/40 (7.5%, 95% CI 1.6%–20.4%) persons (Appendix Table 1).

We extracted DNA by using the Blood/Tissue DNA Kit (MEBEP Bio Science, https://www.mebep.com) for 114 blood samples and guanidine isothiocyanate and phenol/chloroform technique (*7*) for 638 lice (82 pools). We confirmed successful extractions by PCR of glyceraldehyde-3-phosphate dehydrogenase (blood) and invertebrate mitochondrial cytochrome c oxidase subunit I (lice) genes (*8*,*9*). We screened DNA samples for *Bartonella* spp. by PCR of citrate synthase (*gltA*) and β subunit of RNA polymerase (*rpoB*) genes and for *Rickettsia* spp. by PCR of rickettsial 17-kDa antigen gene, as previously described (*10*–*12*). We used ultrapure water as a negative control and genomic DNA from *B*. *henselae* and *R*. *sibirica* as positive controls. A total of 14/82 (17.0%, 95% CI 9.7%–27.0%) louse pools and 1/114 (0.9%, 95% CI 0.02%–4.8%) blood samples were positive for *gltA* and *rpoB* but negative for *Rickettsia* spp. (Appendix Table 2). 

Amplicons were purified and sequenced at the University of Texas Medical Branch (Galveston, TX, USA). The *gltA* and *rpoB* sequences showed 100% identity to *B. quintana* strain NCTC12899 (GenBank accession no. LS483373.1) by BLASTn analysis (https://blast.ncbi.nlm.nih.gov). *B. quintana* sequences generated in this study were deposited in GenBank (accession nos. ON808843 and ON808844). The person whose blood was PCR-positive for *B. quintana* was not infested with body lice but demonstrated high levels of IgG against *B. quintana* (titer >1,024) and TGR (titers were 1,024 for *R. typhi* and 512 for *R. prowazekii*).

We chose risk factor variables by using unconditional logistic regression models (p<0.25) and conditional logistic regression to determine relationships between putative risk factors and serologic status. We used Bayesian information criteria to assess the goodness-of-fit for the models. We used R software version 4.1.2 (The R Project for Statistical Computing, https://www.r-project.org) for all statistical analyses and summarized the final conditional logistic regression model (Table). Although the final model for *Bartonella* spp. revealed 3 variables, only 1 was statistically significant and showed an association between body louse infestation and higher risk for *Bartonella* spp. seropositivity (OR [odds ratio] 2.9, 90% CI 1.1–8.1). The final TGR model contained 5 variables of which 3 were associated with higher seropositivity risk, including self-identifying as white (OR 3.9, 90% CI 1.6–10.7), syphilis seropositivity (OR 3.6, 90% CI 1.5–9.4), and homelessness because of unemployment (OR 2.3, 90% CI 1.02–5.5). We detected 5 variables for combined *Bartonella* spp. and TGR of which 4 variables were associated with seropositivity, including self-identifying as white (OR 5.6, 90% CI 2.2–15.5), monthly change of clothes (OR 0.08, 90% CI 0.07–0.4), homelessness because of family conflicts (OR 0.4, 90% CI 0.2–0.8), and higher total plasma protein (OR 2.0, 90% CI 1.1–4.0).

**Table:** Conditional logistic regression model results showing factors associated with exposure to *Bartonella* spp. and TGR among persons experiencing homelessness in São Paulo, Brazil, June–August 2018*

Model variables	Odds ratio (90% CI)	p value
*Bartonella* spp.†		
Intercept	1.72 (0.86–3.56)	0.20
Ethnicity		
Not white	Referent	NA
White	1.03 (0.30–5.40)	0.97
Body lice infestation		
No	Referent	NA
Yes	2.86 (1.06–8.06)	0.08
Have cats		
No	Referent	NA
Yes	0.24 (0.04–1.10)	0.13
TGR‡		
Intercept	0.47 (0.21–1.02)	0.11
Ethniicity		
Not white	Referent	NA
White	3.94 (1.56–10.67)	0.02
Cause of homelessness: unemployment		
No	Referent	NA
Yes	2.33 (1.02–5.46)	0.09
Frequency of changing clothes		
>Monthly	Referent	NA
Monthly	0.01 (0.001–99.53)	0.99
Syphilis infection		
No	Referent	NA
Yes	3.64 (1.50–9.37)	0.02
Duration of homelessness		
<1 y	Referent	NA
>1 y	0.65 (0.28–1.51)	0.40
Combined *Bartonella* spp. and TGR§		
Intercept	0.001 (0.0001–0.03)	0.02
Ethnicity		
Not white	Referent	NA
White	5.56 (2.18–15.50)	0.004
Total plasma protein	2.01 (1.04–4.00)	0.086
Packed cell volume	1.09 (0.99–1.21)	0.127
Cause of homelessness: family conflicts		
No	Referent	NA
Yes	0.36 (0.16–0.80)	0.038
Frequency of changing clothes		
>Monthly	Referent	NA
Monthly	0.08 (0.07–0.42)	0.03

*Bayesian information criteria (BICs) were used to assess the goodness-of-fit for each model. NA, not applicable; TGR, typhus group rickettsiae. †Model BIC = 100.1. ‡Model BIC = 139.6. §Model BIC = 140.5.

## Conclusions

Our study revealed *Bartonella* spp. and TGR exposure, associated risk factors related to serologic status, and *B. quintana* detection in lice and one blood sample among persons experiencing homelessness in São Paulo, Brazil. Seroprevalence of *Bartonella* spp. (72.5%) was higher in our study than previous reports for persons experiencing homelessness (1.8%–65%) (*13*), and *B. quintana* was the dominant antigen involved in homologous reactions. The highest *B. quintana* seroprevalence was previously found in France (65%, antibody titers >100) and Japan (57%, titers >128) (*13*); those titers were considered indicative of previous exposure. In our study, the antibody titer cutoff was >64, explaining our high seroprevalence results, although all but 2 titers were >128. TGR seropositivity in our study (36.7%) was within the range observed in the United States, Europe, and Colombia (0.54%–56.2%) (*1*,*3*).

Persons experiencing homelessness in São Paulo had *P. humanus humanus* body louse infestation and seropositivity for *B. quintana* and TGR similar to that reported previously (*1*). Body louse infestation (14.9%) was within the range of other reports (7%–22%) (*14*), highlighting global vulnerability to louse infestation and louseborne diseases in persons experiencing homelessness (*1*).

Through logistic regression, we showed seropositivity for *Bartonella* spp. was associated with louse infestation. Because the association of white ethnicity and TRG seropositivity (alone and in combination with *Bartonella* spp.) might be from a skewed population sampling, our findings should be further investigated. Nonetheless, higher TGR seropositivity was associated with homelessness because of unemployment, duration of homelessness, and syphilis seropositivity, which represent risk factors that reflect vulnerability and socioeconomic conditions. In addition, seropositivity for both *Bartonella* and TGR was associated with infrequent changes of clothing.

The first limitation of our study is that the small sample size and power for the examined variables might have weakened associations of *Bartonella* seroreactivity with other variables included in our questionnaire, such as alcoholism, tobacco or intravenous drug use, and homelessness as previously reported (*15*), and variables that were significant in univariate analysis. In addition, IgG seropositivity reflects past *Bartonella* and TGR infections (*6*,*13*). IFA cross-reactivity should be addressed with future studies by using cross-adsorption techniques.

Our results should alert public health professionals in the city of São Paulo to initiate preemptive measures and active vector control among persons experiencing homelessness and confirm circulation of *Bartonella* and TGR species. Clinicians should also consider these vectorborne agents as probable etiologic agents of febrile syndromes in this vulnerable population.

Appendix Additional information for *Bartonella* spp. and typhus group rickettsiae among persons experiencing homelessness in São Paulo, Brazil.
