# Supplementary material for: Bartonella spp. and Typhus Group Rickettsiae among Persons Experiencing Homelessness, São Paulo, Brazil
Source: Emerg Infect Dis. 2023 Feb;29(2):418–21. doi: 10.3201/eid2902.221050 (PMC9881779; doi:10.3201/eid2902.221050)
Supplement: Appendix — Additional information for Bartonella spp. and typhus group rickettsiae among persons experiencing homelessness in São Paulo, Brazil. [file 22-1050-Techapp-s1.pdf]

Article DOI: https://doi.org/10.3201/eid2902.221050

Bartonella spp. and Typhus Group Rickettsiae among Persons Experiencing Homelessness, São Paulo, Brazil

Appendix

Additional Methods

Persons experiencing homelessness responded to a questionnaire that, combined with medical and demographic records, was used to assess risk factors for exposure to *Bartonella* spp. and typhus group rickettsiae in São Paulo city, Brazil, during June–August 2018. The questionnaire was administered in the Brazilian Portuguese language and verbal responses were recorded by a project coordinator.

Questionnaire Information

Demographic variables

- Sample location (diverse locations within São Paulo city, Brazil)

- Neighborhood of sampling (diverse neighborhoods within São Paulo city, Brazil)

- Age (years)

- Sex or gender (male/female)

- Marital status (single/married)

- Self-identified race/ethnicity (white/not white)

- Education (illiterate/literate/not informed)

- Assistance by counselling and psychological services (yes/no)

- Current drug use (yes/no)

- Alcohol consumption (yes/no)

- Tobacco consumption (yes/no)

- Marijuana consumption (yes/no)

- Cocaine consumption (yes/no)

- Crack consumption (yes/no)

- Other drug consumption (drug name)

- City of origin (city in Brazil, city outside Brazil, other country)

- Travel to other cities (yes/no/no response)

- Homelessness duration (months)

- Resting place: shelter/friend's home (yes/no)

- Resting place: on the street (yes/no)

- Resting place: settlement (yes/no)

- Causes for homelessness (yes/no/not informed; following options: lost home; alcohol and drugs, unemployment, family conflicts)

- Has any companion animals? (yes/no/no response)

- Has dog as companion animal? (yes/no/no response)

- Number of dogs that he/she has (number)

- Has cat as companion animal? (yes/no/no response)

- Number of cats that he/she has (number)

- How many people do you live with? (0–2 persons/3–4 persons/>5 persons/no response)

- Frequency of showering (daily/2 times per week/weekly/monthly/no response)

- Do you launder your clothes? (yes/no)

- How do you launder your clothes? (yes/no/not informed; following options: with water, soap and water, soak clothes).

- Do you change your clothes? (daily/2 times per week/weekly/monthly/no response)

- Do you share clothes? (yes/no/no response)

- Do you know what body lice are? (yes/no/no response)

- Have you experienced a previous body louse infestation? (yes/no/no response/not evaluated)

- Have you seen rats? (yes/no/no response/not evaluated)

- Frequency of rat visualization (daily/1 time per week/>1 time per week/not seen/no response/not evaluated)

- Have you experienced a rat bite (yes/no/no response/not evaluated)

- Presence of body lice at the moment of sampling (yes/no)

Medical variables

- Chest pain (yes/no/no response)

- Joint pain (yes/no/no response)

- Eye pain (yes/no/no response)

- Headache (yes/no/no response)

- Difficulty breathing (yes/no/no response)

- Abdominal pain (yes/no/no response)

- Fever for ≥2 weeks with unknown origin (continuous/relapsing/no/no response/not evaluated)

- Autoimmune disease (yes/no/no response)

- Which autoimmune disease? (open response)

- Self-care appearance (yes/no/not evaluated)

- Unpleasant smell present (yes/no/not evaluated)

- Packed cell volume, data obtain from (*1*)

- Total protein, data obtain from (*1*).

- HIV serological status, data obtain from (*2*)

- Syphilis serological status, data obtain from (*2*)

- Hepatitis C serological status, data obtain from (*2*).

References

1. Felipetto LG, Tieder-Junior PI, da Silva FFV, Yamakawa AC, Kmetiuk LB, Couto ACD, et al. Serosurvey of anti-*Toxoplasma gondii* antibodies in homeless persons of São Paulo city, southeastern Brazil. Front Public Health. 2020;8:580637. PubMed https://doi.org/10.3389/fpubh.2020.580637

2. Felipetto LG, Teider-Junior PI, da Silva FFV, Couto ACD, Kmetiuk LB, Martins CM, et al. Serosurvey of anti-*Treponema pallidum* (syphilis), anti-hepatitis C virus and anti-HIV antibodies in homeless persons of São Paulo city, southeastern Brazil. Braz J Infect Dis. 2021;25:101602. PubMed https://doi.org/10.1016/j.bjid.2021.101602

Appendix Table 1. Titers of IgG against *Bartonella* and typhus group rickettsiae in 109 blood samples from persons experiencing homelessness in São Paulo, Brazil, during June–August 2018*

Sample†	*B. quintana*	*B. machadoae*	*B. henselae*	*R. typhi*	*R. prowazekii*	Interpretation
SP01	512	<64	<64	<64	<64	*Bartonella* spp. seroreactive; PAIHR, *B. quintana*
SP02	<64	<64	<64	<64	<64	Not seroreactive to *Bartonella* spp. or TGR
SP03	<64	<64	<64	<64	<64	Not seroreactive to *Bartonella* spp. or TGR
SP05	256	<64	<64	<64	<64	*Bartonella* spp. seroreactive; PAIHR, *B. quintana*
SP11	256	<64	<64	<64	<64	*Bartonella* spp. seroreactive; PAIHR, *B. quintana*
SP14	256	<64	<64	<64	<64	*Bartonella* spp. seroreactive; PAIHR, *B. quintana*
SP16	512	<64	<64	<64	<64	*Bartonella* spp. seroreactive; PAIHR, *B. quintana*
SP17	512	<64	<64	128	128	*Bartonella* and TGR seroreactive; PAIHR, *B. quintana*
SP18	256	<64	<64	<64	<64	*Bartonella* spp. seroreactive; PAIHR, *B. quintana*
SP19	512	<64	<64	<64	<64	*Bartonella* spp. seroreactive; PAIHR, *B. quintana*
SP20	256	<64	<64	<64	<64	*Bartonella* spp. seroreactive; PAIHR, *B. quintana*
SP21	128	<64	<64	128	128	*Bartonella* and TGR seroreactive; PAIHR, *B. quintana*
SP24	512	<64	<64	256	256	*Bartonella* and TGR seroreactive; PAIHR, *B. quintana*
SP25	≥1024	<64	<64	<64	<64	*Bartonella* spp. seroreactive; PAIHR, *B. quintana*
SP26	≥1024	<64	<64	512	512	*Bartonella* and TGR seroreactive; PAIHR, *B. quintana*
SP27	≥1024	<64	<64	<64	<64	*Bartonella* spp. seroreactive; PAIHR, *B. quintana*
SP28	≥1024	<64	<64	<64	<64	*Bartonella* spp. seroreactive; PAIHR, *B. quintana*
SP29	≥1024	<64	<64	512	512	*Bartonella* and TGR seroreactive; PAIHR, *B. quintana*
SP30	<64	<64	<64	<64	<64	Not seroreactive to *Bartonella* spp. or TGR
SP31	256	<64	<64	<64	<64	*Bartonella* spp. seroreactive; PAIHR, *B. quintana*
SP33	<64	<64	<64	<64	<64	Not seroreactive to *Bartonella* spp. or TGR
SP34	512	<64	<64	128	128	*Bartonella* and TGR seroreactive; PAIHR, *B. quintana*
SP35	256	64	64	<64	128	*Bartonella* and TGR seroreactive; PAIHR, *B. quintana* and *R. prowazekii*
SP36	512	<64	<64	<64	<64	*Bartonella* spp. seroreactive; PAIHR, *B. quintana*
SP37	256	<64	<64	<64	<64	*Bartonella* spp. seroreactive; PAIHR, *B. quintana*
SP38	≥1024	64	<64	≥1024	≥1024	*Bartonella* and TGR seroreactive; PAIHR, *B. quintana*
SP39	≥1024	<64	<64	512	512	*Bartonella* and TGR seroreactive; PAIHR, *B. quintana*
SP40	256	<64	<64	<64	<64	*Bartonella* spp. seroreactive; PAIHR, *B. quintana*
SP41	≥1024	<64	<64	512	≥1024	*Bartonella* and TGR seroreactive; PAIHR, *B. quintana* and *R. prowazekii*
SP42	256	<64	<64	128	128	*Bartonella* and TGR seroreactive; PAIHR, *B. quintana*
SP43	64	<64	<64	<64	<64	*Bartonella* spp. seroreactive
SP44	≥1024	64	64	128	64	*Bartonella* and TGR seroreactive; PAIHR, *B. quintana*

Sample†	*B. quintana*	*B. machadoae*	*B. henselae*	*R. typhi*	*R. prowazekii*	Interpretation
SP45	<64	<64	<64	<64	<64	Not seroreactive to *Bartonella* spp. or TGR
SP46	≥1024	<64	<64	512	256	*Bartonella* and TGR seroreactive; PAIHR, *B. quintana*
SP47	≥1024	64	64	<64	<64	*Bartonella* spp. seroreactive; PAIHR, *B. quintana*
SP48	128	64	64	<64	<64	*Bartonella* spp. seroreactive
SP49	128	<64	<64	<64	<64	*Bartonella* spp. seroreactive; PAIHR, *B. quintana*
SP50	<64	<64	<64	<64	<64	Not seroreactive to *Bartonella* spp. or TGR
SP51	≥1024	<64	<64	<64	<64	*Bartonella* spp. seroreactive; PAIHR, *B. quintana*
SP52	<64	<64	<64	<64	<64	Not seroreactive to *Bartonella* spp. or TGR
SP53	<64	<64	<64	<64	<64	Not seroreactive to *Bartonella* spp. or TGR
SP54	<64	<64	<64	<64	<64	Not seroreactive to *Bartonella* spp. or TGR
SP55	<64	<64	<64	<64	<64	Not seroreactive to *Bartonella* spp. or TGR
SP56	≥1024	<64	<64	<64	<64	*Bartonella* spp. seroreactive; PAIHR, *B. quintana*
SP57	≥1024	<64	<64	256	64	*Bartonella* and TGR seroreactive; PAIHR, *B. quintana* and *R. typhi*
SP58	<64	<64	<64	64	<64	TGR seroreactive
SP59	<64	<64	<64	64	<64	TGR seroreactive
SP60	<64	<64	64	64	<64	*Bartonella* and TGR seroreactive
SP61	<64	<64	<64	<64	<64	Not seroreactive to *Bartonella* spp. or TGR
SP62	512	<64	<64	<64	<64	*Bartonella* spp. seroreactive; PAIHR, *B. quintana*
SP63	512	<64	<64	<64	<64	*Bartonella* spp. seroreactive; PAIHR, *B. quintana*
SP64	<64	<64	<64	<64	<64	Not seroreactive to *Bartonella* spp. or TGR
SP65	<64	<64	<64	<64	<64	Not seroreactive to *Bartonella* spp. or TGR
SP66	<64	<64	<64	<64	<64	Not seroreactive to *Bartonella* spp. or TGR
SP67	≥1024	<64	<64	≥1024	512	*Bartonella* and TGR seroreactive; PAIHR, *B. quintana* and *R. typhi*
SP68	≥1024	64	64	<64	<64	*Bartonella* spp. seroreactive; PAIHR, *B. quintana*
SP69	512	64	256	256	256	*Bartonella* and TGR seroreactive
SP70	≥1024	<64	<64	<64	<64	*Bartonella* spp. seroreactive; PAIHR, *B. quintana*
SP71	512	<64	<64	≥1024	256	*Bartonella* and TGR seroreactive; PAIHR, *B. quintana* and *R. typhi*
SP72	≥1024	<64	<64	512	128	*Bartonella* and TGR seroreactive; PAIHR, *B. quintana* and *R. typhi*
SP73	512	<64	<64	<64	<64	*Bartonella* spp. seroreactive; PAIHR, *B. quintana*
SP74	<64	<64	<64	<64	128	TGR seroreactive; PAIHR,*R. prowazekii*
SP75	≥1024	<64	<64	<64	<64	*Bartonella* spp. seroreactive; PAIHR, *B. quintana*
SP76	128	<64	<64	<64	<64	*Bartonella* spp. seroreactive; PAIHR, *B. quintana*
SP77	256	<64	<64	<64	<64	*Bartonella* spp. seroreactive; PAIHR, *B. quintana*
SP78	<64	<64	<64	<64	<64	Not seroreactive to *Bartonella* spp. or TGR
SP79	512	<64	<64	256	64	*Bartonella* and TGR seroreactive; PAIHR, *B. quintana* and *R. typhi*
SP80	≥1024	64	64	64	<64	*Bartonella* and TGR seroreactive; PAIHR, *B. quintana*
SP81	256	<64	<64	64	64	*Bartonella* and TGR seroreactive; PAIHR, *B. quintana*
SP82	512	<64	<64	<64	<64	*Bartonella* spp. seroreactive; PAIHR, *B. quintana*
SP83	≥1024	<64	<64	128	128	*Bartonella* and TGR seroreactive; PAIHR, *B. quintana*
SP84	64	<64	<64	<64	<64	*Bartonella* sp. seroreactive
SP85	<64	<64	<64	<64	<64	Not seroreactive to *Bartonella* spp. or TGR
SP86	<64	<64	<64	<64	64	TGR seroreactive
SP87	≥1024	<64	<64	256	128	*Bartonella* and TGR seroreactive; PAIHR, *B. quintana*
SP88	≥1024	<64	<64	256	64	*Bartonella* and TGR seroreactive; PAIHR, *B. quintana* and *R. typhi*
SP89	512	<64	<64	≥1024	512	*Bartonella* and TGR seroreactive; PAIHR, *B. quintana* and *R. typhi*
SP90	<64	<64	<64	<64	<64	Not seroreactive to *Bartonella* spp. or TGR
SP91	<64	<64	<64	<64	<64	Not seroreactive to *Bartonella* spp. or TGR
SP92	≥1024	<64	<64	<64	<64	*Bartonella* spp. seroreactive; PAIHR, *B. quintana*
SP93	<64	<64	<64	<64	<64	Not seroreactive to *Bartonella* spp. or TGR
SP94	512	<64	<64	<64	<64	*Bartonella* spp. seroreactive; PAIHR, *B. quintana*
SP95	256	<64	<64	<64	<64	*Bartonella* spp. seroreactive; PAIHR, *B. quintana*
SP96	<64	<64	<64	<64	<64	Not seroreactive to *Bartonella* spp. or TGR
SP97	<64	<64	<64	<64	<64	Not seroreactive to *Bartonella* spp. or TGR
SP99	≥1024	<64	<64	≥1024	512	*Bartonella* and TGR seroreactive; PAIHR, *B. quintana* and *R. typhi*
SP100	512	<64	<64	<64	<64	*Bartonella* spp. seroreactive; PAIHR, *B. quintana*
SP101	<64	<64	<64	<64	<64	Not seroreactive to *Bartonella* spp. or TGR

Sample†	*B. quintana*	*B. machadoae*	*B. henselae*	*R. typhi*	*R. prowazekii*	Interpretation
SP102	256	<64	64	<64	<64	*Bartonella* spp. seroreactive; PAIHR, *B. quintana*
SP103	≥1024	<64	<64	<64	<64	*Bartonella* spp. seroreactive; PAIHR, *B. quintana*
SP104	256	64	<64	≥1024	512	*Bartonella* and TGR seroreactive; PAIHR, *B. quintana* and *R. typhi*
SP105	256	<64	<64	<64	64	*Bartonella* and TGR seroreactive; PAIHR, *B. quintana*
SP106	256	<64	<64	<64	<64	*Bartonella* spp. seroreactive; PAIHR, *B. quintana*
SP107	≥1024	<64	<64	≥1024	512	*Bartonella* and TGR seroreactive; PAIHR, *B. quintana* and *R. typhi*
SP108	≥1024	<64	<64	64	64	*Bartonella* and TGR seroreactive; PAIHR, *B. quintana*
SP109	512	<64	<64	256	64	*Bartonella* and TGR seroreactive; PAIHR, *B. quintana* and *R. typhi*
SP110	<64	<64	<64	<64	<64	Not seroreactive to *Bartonella* spp. or TGR
SP111	128	<64	<64	<64	<64	*Bartonella* spp. seroreactive; PAIHR, *B. quintana*
SP112	<64	<64	<64	<64	<64	Not seroreactive to *Bartonella* spp. or TGR
SP113	≥1024	<64	64	256	64	*Bartonella* and TGR seroreactive; PAIHR, *B. quintana* and *R. typhi*
SP114	256	<64	<64	<64	<64	*Bartonella* spp. seroreactive; PAIHR, *B. quintana*
SP115	≥1024	<64	<64	128	64	*Bartonella* and TGR seroreactive; PAIHR, *B. quintana*
SP 116	≥1024	64	<64	256	128	*Bartonella* and TGR seroreactive; PAIHR, *B. quintana*
SP118	≥1024	<64	<64	128	64	*Bartonella* and TGR seroreactive; PAIHR, *B. quintana*
SP119	<64	<64	<64	256	<64	TGR seroreactive; PAIHR, *R. typhi*
SP120	512	<64	<64	512	256	*Bartonella* and TGR seroreactive; PAIHR, *B. quintana*
SP121	<64	<64	<64	<64	<64	Not seroreactive to *Bartonella* spp. or TGR
SP122	512	<64	<64	128	64	*Bartonella* and TGR seroreactive; PAIHR, *B. quintana*
SP123	≥1024	<64	<64	512	256	*Bartonella* and TGR seroreactive; PAIHR, *B. quintana*

*IgG titers were determined by indirect immunofluorescence assay with commercial slides for *Bartonella quintana* (12-well IFA Substrate Slides, Fuller Laboratories, http://www.fullerlaboratories.com) and in-house slides for *B. henselae* ST9, *B. machadoae* 56A, *Rickettsia typhi* Galveston, and *Rickettsia prowazekii* Breinl strains. PAIHR, possible antigen involved in a homologous reaction; TGR, typhus group rickettsiae.
†Serum samples were not available for SP07, SP22, and SP32.

Appendix Table 2. Detection of *Bartonella* and *Rickettsia* spp. DNA in lice collected from persons (n = 17) experiencing homelessness in São Paulo, Brazil, during June–August 2018*

Sample ID*	Body louse pool ID	Composition of body louse pools	*Bartonella* spp./*Rickettsia* spp. DNA
SP01	2	4 males	Negative/Negative
	3	10 nymphs	Negative/Negative
	4	8 nymphs	Negative/Negative
SP07	6	1 female	Negative/Negative
	7	10 nymphs	Negative/Negative
	8	10 nymphs	Negative/Negative
	9	10 nymphs	Negative/Negative
	10	10 nymphs	Negative/Negative
	11	8 nymphs	Negative/Negative
SP19	No DNA extraction	NA	NA
SP22	13	1 male	Negative/Negative
	15	1 nymph	Negative/Negative
SP26	16	1 male	Negative/Negative
SP27	18	6 males	Negative/Negative
	19	6 males	Negative/Negative
	21	6 females	Negative/Negative
	22	10 nymphs	Negative/Negative
	23	10 nymphs	Negative/Negative
	24	10 nymphs	Negative/Negative
	25	10 nymphs	Negative/Negative
	26	10 nymphs	Negative/Negative
	27	10 nymphs	Negative/Negative
	29	10 nymphs	Negative/Negative
	30	10 nymphs	Negative/Negative
	31	10 nymphs	Negative/Negative
	32	10 nymphs	Negative/Negative

Sample ID*	Body louse pool ID	Composition of body louse pools	*Bartonella* spp./*Rickettsia* spp. DNA
	33	10 nymphs	Negative/Negative
	34	10 nymphs	Negative/Negative
	35	10 nymphs	Negative/Negative
	36	10 nymphs	Negative/Negative
	37	10 nymphs	Negative/Negative
	38	10 nymphs	Negative/Negative
	39	10 nymphs	Negative/Negative
	40	10 nymphs	Negative/Negative
	41	10 nymphs	Negative/Negative
	42	10 nymphs	Negative/Negative
	43	10 nymphs	Negative/Negative
	44	10 nymphs	Negative/Negative
	45	10 nymphs	Negative/Negative
	46	10 nymphs	Negative/Negative
	47	10 nymphs	Negative/Negative
	48	10 nymphs	Negative/Negative
	49	10 nymphs	Negative/Negative
	50	10 nymphs	Negative/Negative
	51	10 nymphs	Negative/Negative
	52	10 nymphs	Negative/Negative
	53	10 nymphs	Negative/Negative
	54	10 nymphs	Negative/Negative
	56	10 nymphs	Negative/Negative
SP31	58	6 nymphs	Negative/Negative
SP32	60	4 females	Negative/Negative
	61	6 nymphs	Negative/Negative
SP47	No DNA extraction	NA	NA
SP65	65	2 males	Negative/Negative
	67	6 nymphs	Negative/Negative
	68	5 nymphs	Negative/Negative
SP75	No DNA extraction	NA	NA
SP79	No DNA extraction	NA	NA
SP80	71	5 males	Positive/Negative
	72	5 males	Positive/Negative
	73	5 males	Negative/Negative
	74	5 males	Positive/Negative
	75	5 males	Positive/Negative
	76	5 males	Negative/Negative
	77	5 males	Positive/Negative
	79	5 males	Positive/Negative
	80	4 males	Positive/Negative
	83	5 females	Positive/Negative
	84	5 females	Positive/Negative
	85	5 females	Positive/Negative
	86	5 females	Positive/Negative
	87	5 females	Positive/Negative
	88	5 females	Negative/Negative
	89	5 females	Positive/Negative
	90	5 females	Negative/Negative
	91	3 females	Negative/Negative
	93	10 nymphs	Negative/Negative
	94	10 nymphs	Negative/Negative
	96	10 nymphs	Negative/Negative
	97	10 nymphs	Negative/Negative
	98	10 nymphs	Negative/Negative
	99	10 nymphs	Negative/Negative
	100	10 nymphs	Negative/Negative
	101	10 nymphs	Negative/Negative
	102	10 nymphs	Negative/Negative
	103	10 nymphs	Positive/Negative
SP101	No DNA extraction	NA	NA
SP108	No DNA extraction	NA	NA
SP109	No DNA extraction	NA	NA
SP113	No DNA extraction	NA	NA

*We screened DNA samples for *Bartonella* spp. by PCR of citrate synthase (*gltA*) and β subunit of RNA polymerase (*rpoB*) genes and for *Rickettsia* spp. by PCR of rickettsial 17-kDa antigen gene. Results for antibodies against *Bartonella* and typhus group rickettsiae are in Appendix Table 1. ID, identification; NA, not applicable.
